# Time Trends in Hospital Admissions for Bronchiectasis: Analysis of the Spanish National Hospital Discharge Data (2004 to 2013)

**DOI:** 10.1371/journal.pone.0162282

**Published:** 2016-09-13

**Authors:** Gema Sánchez-Muñoz, Ana López de Andrés, Rodrigo Jiménez-García, Pilar Carrasco-Garrido, Valentín Hernández-Barrera, Fernando Pedraza-Serrano, Luis Puente-Maestu, Javier de Miguel-Díez

**Affiliations:** 1 Respiratory Department, Hospital General Universitario Gregorio Marañón, Facultad de Medicina, Universidad Complutense de Madrid (UCM), Instituto de Investigación Sanitaria Gregorio Marañón (IiSGM), Madrid, Spain; 2 Preventive Medicine and Public Health Teaching and Research Unit, Department of Health Sciences, Universidad Rey Juan Carlos, Alcorcón, Madrid, Spain; University of Western Australia, AUSTRALIA

## Abstract

**Objective:**

To analyze changes in the incidence, diagnostic procedures, comorbidity, length of hospital stay (LOHS), costs and in-hospital mortality (IHM) for patients with bronchiectasis who were hospitalized in Spain over a 10-year period.

**Methods:**

We included all admissions for patients diagnosed with bronchiectasis as primary or secondary diagnosis during 2004–2013.

**Results:**

282,207 patients were admitted to the study. After controlling for possible confounders, we observed a significant increase in the incidence of hospitalizations over the study period when bronchiectasis was a secondary diagnosis. When bronchiectasis was the primary diagnosis we observed a significant decline in the incidence. In all cases, this pathology was more frequent in males, and the average age and comorbidity increased significantly during the study period (p<0.001). When bronchiectasis was the primary diagnosis, the most frequent secondary diagnosis was Pseudomonas aeruginosa infection. When bronchiectasis was the secondary diagnosis, the most frequent primary diagnosis was COPD. IHM was low, tending to decrease from 2004 to 2013 (p<0.05). The average LOHS decreased significantly during the study period in both cases (p<0.001). The mean cost per patient decreased in patients with bronchiectasis as primary diagnosis, but it increased for cases of bronchiectasis as secondary diagnosis (p<0.001).

**Conclusions:**

Our results reveal an increase in the incidence of hospital admissions for patients with bronchiectasis as a secondary diagnosis from 2004 to 2013, as opposed to cases of bronchiectasis as the primary diagnosis. Although the average age and comorbidity significantly increased over time, both IHM and average LOHS significantly decreased.

## Introduction

Bronchiectasis is characterized by abnormal and irreversible bronchial dilation, with ciliary epithelium dysfunction as a consequence of clinical pathology that includes respiratory infection, chronic inflammation and mucociliary system lesions [1]. Since it was first described in 1819 by Laennec, the approach and treatment of bronchiectasis have experienced significant changes, which have led to a decrease in morbidity and mortality. Bronchiectasis even came to be considered a disease on the verge of extinction. In fact, Barker described this pathology as “the orphan disease” in a review published in the 80s, due to the scarce scientific and commercial interest it then inspired [2]. However, less severe but more frequent forms are currently being diagnosed. This could be explained principally by the high resolution images produced by computerized tomography (CT) techniques, increased population longevity, and increased chronicity of disease [3]. The true prevalence of bronchiectasis is not precisely known and, it may vary significantly from one country to another. In the USA, a prevalence of 52.3 cases per 100,000 adults has been estimated, with greater incidence in women and the elderly [4]. However, in Finland, incidence is 2.7 per 100,000 inhabitants [5]. Among the infantile population of New Zealand, the rate is 3.7 per 100,000 inhabitants, although wide variations are seen, depending on ethnic origin [6].

Bronchiectasis constitutes an important health concern. According to data reported by the UK Department of Health more than a decade ago, up to 78% of patients who visited the ER required admission, with an average stay greater than 10 days, thus higher than had been estimated for other disease processes such as COPD [7]. In recent years, more studies have been published intended to quantify the socio-healthcare impact of bronchiectasis [8–11]. Particularly notable among these is the study carried out by Seitz et al [8]. They describe a hospitalization rate, adjusted for age, in the USA from 1993 to 2006, of approximately 16.5 per 100,000 inhabitants. The rate was higher for women and the elderly, and was observed to increase during the study period.

Data collection on hospitalization admissions for bronchiectasis at the national level is important in order to assess incidence, patient characteristics, and results in terms of several variables such as mean hospital stay, cost, morbidity and mortality. There are no Spanish epidemiological studies available for this pathology, although national registries are currently being created for several respiratory diseases, including bronchiectasis, which may throw light on the situation in the future [12].

Comparing hospital admissions and outcomes for bronchiectasis between countries could help each country better understand their own data and could also aid in making provisions for future healthcare services. The National Hospital Discharge Database provides a large alternative information source to describe and analyze the trends and characteristics of hospitalization for bronchiectasis at the national level.

The aim of this study was to conduct a nationwide analysis of discharge data, collected from 2004 to 2013 years. These data were used to elucidate changes in the incidence, diagnostic procedures, comorbidity profiles, length of hospital stay (LOHS), economic costs and in-hospital mortality (IHM) for patients with bronchiectasis who were hospitalized in Spain over a 10-year study period.

## Methods

According to the Spanish Health System, at the time of discharge after each hospital stay physicians must report all diagnoses and procedures performed, using the International Classification for Disease, 9th revision codes (ICD-9-CM). This information is collected by the Spanish National Hospital Database, the *Conjunto Mínimo Basico de Datos* (CMBD), which compiles all hospital data for the Spanish National Health System [13]. The CMBD database includes patient variables (gender and date of birth), date of admission, date of discharge, discharge destination (home, other healthcare/nursing facility, morgue), and includes details for up to 14 discharge diagnoses and up to 20 procedures performed during admission.

We selected all admissions for patients diagnosed with bronchiectasis as the primary or secondary diagnosis during 2004–2013 (ICD-9-CM 494). We made a separate analysis of bronchiectasis as the primary diagnosis and as a secondary diagnosis. We calculated the annual incidence ratio by dividing the number of cases per year by the corresponding number of people in that population according to the National Institute of Statistics as reported on December 31st each year [14]. The incidence ratios were expressed per 100,000 inhabitants. The proportion of patients who died during the hospital stay (in-hospital mortality), LOHS, and costs were also estimated for each year studied. Costs were calculated using Diagnosis-Related Groups (GRD, *Grupos Relacionados con el Diagnóstico*) for the disease. GRD represents a medical economic entity for a set of diseases requiring analogous management resources [15]. All costs shown were adjusted for inflation during the same period in Spain. Clinical characteristics included information on overall comorbidity at the time of hospitalization, which was assessed using the Charlson comorbidity index [16]. The index applies to 17 disease categories that are totaled to obtain an overall score for each patient. We divided our patients into three categories: 1) low index, which corresponded to patients with no previously recorded disease categories in the Charlson comorbidity index; 2) medium index, patients with one or two disease categories; and 3) high index, patients with more than two disease categories. We specifically identified the following procedures: (CT) computed tomography pulmonary angiography, bronchoscopy (codes 33.21, 33.22, 33.23 and 33.24) and the use of mechanical ventilation treatment during the hospital stay. The use of non-invasive ventilation (NIV) and invasive mechanical ventilation (IMV) was determined based on procedure codes 93.90 and 96.04 respectively.

### Statistical analysis

A descriptive statistical analysis was performed. Quantitative variables were expressed as mean ±SD. Qualitative variables were expressed as frequencies and percentages. Comparisons were performed using the Chi-squared test, Fisher’s exact test, t-test or ANOVA, as appropriate. Multivariate time trend analysis for study variables was conducted using Poisson regression models for incidence, and logistic regression models for in-hospital mortality and age, gender and other co-variable adjustments. Year was included in the model as a continuous variable. So an IRR less or over 1 (with 95% CI not including 1) means that over the study period (2004 to 2013) the incidence of bronchiectasis as primary or secondary diagnosis in Spain has decreased or increased respectively.

We then checked for interactions between the independent variables in the regression models. Estimates were made using STATA version 10.1 (StataCorp LP, College Station, TX, USA); a two-tailed statistical significance level was set at 0.05.

In our study we applied joinpoint log lineal regression to identify the years in which changes in tendency occurred in hospital admission rates for bronchiectasis as primary or secondary diagnosis, as well as to estimate the annual percentage of change (APC) in each of the periods delimited by the points of change. The analysis started with the minimum number of joinpoints and tested whether the inclusion of one or more joinpoints were statistically significant [17]. In the final model, each joinpoint indicated a significant change in tendency, and the APC was obtained in each of the segments delimited by the joinpoints by means of weighted least squares. The Joinpoint Regression Program, version 4.0.4 was used for the analysis [18].

Data were treated with full confidentiality according to Spanish legislation. Patient identifiers were removed before the database was provided to the authors in order to keep strict patient confidentiality. It was not possible to identify individual patients in this study or in the database. Given the anonymous and mandatory nature of the dataset, it was not necessary to obtain informed consent. The Spanish Ministry of Health evaluated our research protocols research and determined that the anonymous database we were provided with met all ethical requirements according to Spanish legislation. Because of all the study aspects given above, further compliance with requirements for ethical approval was not necessary. According to the Spanish legislation

## Results

In all, we identified 282,207 patients with bronchiectasis who were hospitalized in Spain from 2004 to 2013. Of these, 70,676 admissions corresponded to patients with bronchiectasis as the primary diagnosis at discharge (39,680 men and 30,996 women), while for the other 211,531 cases, bronchiectasis was a secondary diagnosis (130,722 men and 80,809 women).

Tables 1 and 2 show the incidence, distribution per age and gender, and characteristics of the patients admitted to the hospital with bronchiectasis as the primary and secondary diagnosis, respectively, for the period 2004–2013 in Spain.

**Table 1:** Incidence and characteristics of patients discharged with a primary diagnosis of bronchiectasis in Spain, 2004–2013.

	Year	2004	2005	2006	2007	2008	2009	2010	2011	2012	2013	Total
Gender, n (%) p = 0.037	Male	3899(55.08)	4229(55.95)	3287(56.08)	3833(56.33)	3765(57.48)	3952(56.7)	3922(56.45)	4071(56.91)	4391(56.05)	4331(54.7)	39680(56.14)
	Female	3180(44.92)	3329(44.05)	2574(43.92)	2971(43.67)	2785(42.52)	3018(43.3)	3026(43.55)	3083(43.09)	3443(43.95)	3587(45.3)	30996(43.86)
Age, years p<0.001	Mean (SD)	71.52(13.96)	72(13.93)	71.54(14.55)	72.41(14.14)	72.23(14.38)	72.36(14.33)	72.83(13.81)	72.8(14.19)	73.95(13.62)	73.49 (13.68)	72.55(14.06)
Age groups, n (%) p<0.001	0–49 years	524(7.4)	538(7.12)	490(8.36)	519(7.63)	496(7.57)	531(7.62)	476(6.85)	519(7.25)	463(5.91)	482 (6.09)	5038(7.13)
	50–64 years	960(13.56)	1004(13.28)	783(13.36)	842(12.38)	903(13.79)	954(13.69)	939(13.51)	936(13.08)	952(12.15)	1016 (12.83)	9289(13.14)
	65–79 years	3581(50.59)	3648(48.27)	2758(47.06)	3212(47.21)	2944(44.95)	3152(45.22)	3159(45.47)	3169(44.3)	3409(43.52)	3445 (43.51)	32477(45.95)
	≥ 80 years	2014(28.45)	2368(31.33)	1830(31.22)	2231(32.79)	2207(33.69)	2333(33.47)	2374(34.17)	2530(35.36)	3010(38.42)	2975 (37.57)	23872(33.78)
Tobacco use, n (%) p<0.001	Yes	1661(23.46)	1831(24.23)	1374(23.44)	1621(23.82)	1672(25.53)	1862(26.71)	1954(28.12)	2014(28.15)	2294(29.28)	2332 (29.45)	18615(26.34)
Charlson index, n (%) p<0.001	0	4195(59.26)	4330(57.29)	3418(58.32)	3846(56.53)	3542(54.08)	3850(55.24)	3702(53.28)	3722(52.03)	3857(49.23)	3904 (49.31)	38366(54.28)
	1	2137(30.19)	2335(30.89)	1745(29.77)	2084(30.63)	2078(31.73)	2158(30.96)	2209(31.79)	2356(32.93)	2636(33.65)	2651 (33.48)	22389(31.68)
	≥2	747(10.55)	893(11.82)	698(11.91)	874(12.85)	930(14.2)	962(13.8)	1037(14.93)	1076(15.04)	1341(17.12)	1363 (17.21)	9921(14.04)
Pseudomonas aeruginosa infection,n (%) p = 0.008	Yes	987(13.94)	1094(14.47)	927(15.82)	1087(15.98)	1148(17.53)	1260(18.08)	1364(19.63)	1360(19.01)	1509(19.26)	1541 (19.46)	12277(17.37)
Invasive mechanical ventilation, n (%) p<0.001	Yes	53(0.75)	72(0.95)	33(0.56)	34(0.5)	39(0.6)	41(0.59)	23(0.33)	35(0.49)	39(0.5)	42(0.53)	411(0.58)
Non-invasive mechanical ventilation, n (%) p<0.001	Yes	115(1.62)	123(1.63)	82(1.4)	109(1.6)	130(1.98)	149(2.14)	206(2.96)	240(3.35)	286(3.65)	333(4.21)	1773(2.51)
Thoracic computed tomography, n (%) p<0.001	Yes	1148(16.22)	1193(15.78)	1038(17.71)	1194(17.55)	1285(19.62)	1427(20.47)	1434(20.64)	1457(20.37)	1532(19.56)	1620 (20.46)	13328(18.86)
Bronchoscopy, n (%) p = 0.013	Yes	454(6.41)	422(5.58)	398(6.79)	394(5.79)	375(5.73)	466(6.69)	408(5.87)	418(5.84)	444(5.67)	499(6.3)	4278(6.05)
In-hospital mortality, n (%) p<0.001	Yes	265(3.74)	334(4.42)	180(3.07)	225(3.31)	201(3.07)	213(3.06)	221(3.18)	232(3.24)	274(3.5)	261(3.3)	2406(3.4)
Length of stay, days p<0.001	Mean (SD)	10.59(9.27)	10.57(9.47)	10.02(8.4)	10.12(9.65)	9.9(8.12)	9.92(8.67)	9.85(7.93)	9.52(7.84)	9.49(7.55)	9.38(8.48)	9.93(8.57)
Cost, euros p<0.001	Mean (SD)	3960.51 (2721.22)	3798.68 (2567.26)	3565.83 (2207.73)	3705.39 (2362.37)	3960.54 (2418.76)	4007.02 (2703.12)	3602.97 (2838.87)	3766.74 (3068.6)	3595 (2993.29)	3515.39 (3319.26)	3745.36 (2766.22)
Total p<0.001	N	7079	7558	5861	6804	6550	6970	6948	7154	7834	7918	70676
	Incidence^+^	16.52	17.31	13.21	15.04	14.24	15.03	14.92	15.31	16.75	16.99	15.53

^+^ Patients per 100,000 population. P values for time trends.

**Table 2:** Incidence and characteristics of patients discharged with a secondary diagnosis of bronchiectasis in Spain, 2004–2013.

	Year	2004	2005	2006	2007	2008	2009	2010	2011	2012	2013	Total
Gender, n (%) p<0.001	Male	9734(63.23)	10647(62.89)	11281(63.47)	12236(62.13)	12744(61.97)	13592(61.82)	14033(61.72)	14724(61.35)	15432(60.81)	16299(60.2)	130722(61.8)
	Female	5661(36.77)	6283(37.11)	6493(36.53)	7459(37.87)	7821(38.03)	8393(38.18)	8705(38.28)	9276(38.65)	9944(39.19)	10774(39.8)	80809(38.2)
Age, years p<0.001	Mean (SD)	71.23(14.38)	71.58(14.35)	72.03(14.21)	72.29(14.17)	72.35(14.5)	72.49(14.44)	72.94(14.06)	73.29(13.93)	73.69(13.98)	73.7(13.76)	72.69(14.17)
Age groups, n (%) p<0.001	0–49 years	1290(8.38)	1369(8.09)	1326(7.46)	1435(7.29)	1506(7.32)	1606(7.3)	1517(6.67)	1545(6.44)	1540(6.07)	1587(5.86)	14721(6.96)
	50–64 years	2121(13.78)	2279(13.46)	2303(12.96)	2626(13.33)	2774(13.49)	2995(13.62)	3081(13.55)	3147(13.11)	3260(12.85)	3498(12.92)	28084(13.28)
	65–79 years	7511(48.79)	8186(48.35)	8562(48.17)	9188(46.65)	9439(45.9)	9835(44.74)	10024(44.08)	10592(44.13)	10640(41.93)	11514(42.53)	95491(45.14)
	≥ 80 years	4473(29.05)	5096(30.1)	5583(31.41)	6446(32.73)	6846(33.29)	7549(34.34)	8116(35.69)	8716(36.32)	9936(39.16)	10474(38.69)	73235(34.62)
Tobacco use, n (%) p<0.001	Yes	4078(26.49)	4551(26.88)	4853(27.3)	5358(27.2)	5543(26.95)	6018(27.37)	6383(28.07)	6793(28.3)	7046(27.77)	7909(29.21)	58532(27.67)
Charlson index, n (%)<0.001	0	7247(47.07)	7811(46.14)	8022(45.13)	8832(44.84)	9033(43.92)	9370(42.62)	9208(40.5)	9551(39.8)	9543(37.61)	9810(36.24)	88427(41.8)
	1	5328(34.61)	5849(34.55)	6230(35.05)	6938(35.23)	7162(34.83)	7784(35.41)	8260(36.33)	8584(35.77)	9273(36.54)	9943(36.73)	75351(35.62)
	≥2	2820(18.32)	3270(19.31)	3522(19.82)	3925(19.93)	4370(21.25)	4831(21.97)	5270(23.18)	5865(24.44)	6560(25.85)	7320(27.04)	47753(22.57)
Pseudomonas aeruginosa infection,n (%) p = 0.016	Yes	918(5.96)	1017(6.01)	1277(7.18)	1433(7.28)	1633(7.94)	1627(7.4)	1706(7.5)	1683(7.01)	1732(6.83)	1758(6.49)	14784(6.99)
Invasive mechanical ventilation, n (%) p = 0.008	Yes	268(1.74)	251(1.48)	238(1.34)	289(1.47)	284(1.38)	298(1.36)	303(1.33)	296(1.23)	337(1.33)	364(1.34)	2928(1.38)
Non-invasive mechanical ventilation, n (%) p<0.001	Yes	264(1.71)	283(1.67)	433(2.44)	535(2.72)	601(2.92)	749(3.41)	936(4.12)	1053(4.39)	1179(4.65)	1402(5.18)	7435(3.51)
Thoracic computed tomography, n (%) p<0.001	Yes	2575(16.73)	2920(17.25)	2921(16.43)	3477(17.65)	4054(19.71)	4547(20.68)	4892(21.51)	5078(21.16)	5479(21.59)	5971(22.06)	41914(19.81)
Bronchoscopy, n (%) p = 0.002	Yes	833(5.41)	891(5.26)	816(4.59)	905(4.6)	1010(4.91)	1043(4.74)	1144(5.03)	1123(4.68)	1239(4.88)	1286(4.75)	10290(4.86)
In-hospital mortality, n (%) p<0.001	Yes	1043(6.77)	1225(7.24)	1149(6.46)	1374(6.98)	1356(6.59)	1445(6.57)	1491(6.56)	1693(7.05)	1898(7.48)	1747(6.45)	14421(6.82)
Length of stay, days p<0.001	Mean (SD)	11.19(11.33)	11.22(11.22)	10.83(10.87)	10.65(10.29)	10.76(10.81)	10.5(10.69)	10.17(10.37)	9.98(10.81)	9.74(9.96)	9.51(9.45)	10.36(10.53)
Cost p<0.001	Mean (SD)	4326.92 (3478.63)	4207.57 (3476.54)	4117.03 (3500.96)	4341.13 (3815.12)	4731.57 (4008.64)	4757.3 (4087.22)	4638.71 (4656.1)	4823.75 (4759.19)	4733.06 (4853.85)	4558.55 (4471.18)	4553.38 (4227.27)
Total p<0.001	N	15395	16930	17774	19695	20565	21985	22738	24000	25376	27073	211531
	Incidence^+^	35.92	38.77	40.07	43.54	44.72	47.41	48.83	51.35	54.26	58.11	46.48

^+^ Patients per 100,000 population. P values for time trends.

Overall, when we look at bronchiectasis as the principal diagnosis for hospital admission, we observe a slight but significant (p<0.001) increase in incidence- 16.52 to 16.99 admissions per 100,000 inhabitants. This increase was more evident when bronchiectasis corresponded to a secondary diagnosis, increasing from 35.92 to 58.11 per 100,000 inhabitants, (p<0.001). In all cases, this pathology was more common in men, though we observed a trend towards a decrease in the proportion of men for the period studied beside if bronchiectasis was the primary or secondary diagnosis. Average patient age also rose significantly during the study period (p<0.001). Upon analyzing the ratio of cases for each age category, we observed a trend towards a decrease in all ages, except for those patients more than 80 years of age, for whom the number of cases during the study period increased from 28.45% to 37.57% for the group with bronchiectasis as the primary diagnosis, and 29.05% to 38.69% for the group with bronchiectasis as a secondary diagnosis (p<0.001).

The percentage of smokers always remained below 30%, regardless of the primary or secondary position of bronchiectasis although actual tobacco consumption increased from 2004 to 2013 (p<0.001) in both cases. Comorbidity, measured by the Charlson index, in patients admitted to the hospital for bronchiectasis as primary diagnosis varied in a statistically significant manner. In 2004, 59.26% of patients showed an index of 0; 30.19% showed an index of 1; and 10.55% had an index greater or equal to 2. In 2013, the proportion of patients with an index of 1 or higher, or equal to 2, had increased to 33.48% and 17.21%, respectively (p<0.001). A similar and also significant trend was observed when the same analysis was performed on cases of hospital admission for bronchiectasis as a secondary diagnosis. The percentage of patients with a Charlson index of 0 decreased (from 47.07% in 2004 to 36.24% in 2013), while the proportion of patients with an index of 1 increased (from 34.61% in 2004 to 36.73% in 2013), as did the proportion of patients with an index of equal to or greater than 2 (from18.32% in 2004 to 27.04% in 2013). The number of Pseudomonas infections, measured using the ICD-9 482.1 code, increased significantly during the study period. The increase was from 13.9% to 19.46% when bronchiectasis was the main reason for hospital admission (p = 0.008), and from 5.96% to 6.49%, when it was only a secondary diagnosis (p = 0.016). The percentage of cases for which invasive mechanical ventilation was required dropped significantly during the study period, regardless of the diagnosis position. On the other hand, the use of non-invasive mechanical ventilation increased (p<0.001).

For our analysis, we evaluated the use of two diagnostic procedures during the hospital admission process: thoracic CT scan and bronchoscopy. The percentage of cases for which a thoracic CT was performed increased during the study period: from 16.22% in 2004 to 20.46% in 2013, when bronchiectasis was the cause of admission (p<0.001), and from 16.73% in 2004 to 22.06% in 2013, in all other cases (p<0.001). However, the number of bronchoscopies showed a statistically significant decrease in primary and secondary position (p = 0.013 and p = 0.002 respectively).

In-hospital mortality was generally low (3.4% when bronchiectasis was the primary diagnosis, and 6.82% when it was included in a secondary diagnosis). Furthermore, the mortality rate fell from 2004 to 2013, in both situations (p<0.001). The average hospital stay was 10.59 days in 2004, dropping to 9.38 days in 2013 when bronchiectasis was a primary diagnosis (p<0.001). A decrease was also observed in the average stay when bronchiectasis was a secondary diagnosis, falling from 11.19 days in 2004 to 9.51 days in 2013 (p<0.001). The mean cost per patient decreased from 3,960.51 Euros in 2004 to 3,515.39 Euros in 2013 for the group with bronchiectasis as the primary diagnosis, (p<0.001). However, for all other cases, the average cost increased from 4,326.98 Euros to 4,558.55 Euros, (p<0.001)

Table 3 shows the most common secondary diagnoses, when bronchiectasis was the principal reason for admission.

**Table 3:** Most common secondary diagnoses for patients discharged with a primary diagnosis of bronchiectasis in Spain, 2004–2013.

Diagnosis	n	%
Bacterial infection in conditions classified elsewhere and of unspecified site. Pseudomonas	8837	12.5
Other diseases of respiratory system, not elsewhere classified	4551	6.4
Diabetes mellitus	3407	4.8
Essencial hypertension unspecified	2644	3.7
Acute respiratory failure	2556	3.6
Late effects of tuberculosis	2528	3.6
Acute and chronic respiratory failure	2055	2.9
Bacterial infection in conditions classified elsewhere and of unspecified site. Bacterial infection, unspecified	1574	2.2
Tobacco use disorder	1204	1.7
Bacterial infection in conditions classified elsewhere and of unspecified site. Methicillin susceptible Staphylococcus aureus	886	1.3
Hemoptysis	824	1.2
Bacterial infection in conditions classified elsewhere and of unspecified site. Escherichia coli	815	1.2

The most frequent secondary diagnosis was Pseudomonas infection, accounting for 12.5% of admissions. Although less common than Pseudomonas, infections by other microorganisms were also observed, mainly methicillin-sensitive Staphylococcus aureus. Notable non-infectious risk factors for cardiovascular disease included diabetes mellitus, hypertension, tuberculosis sequelae, and respiratory disorders, both acute and chronic.

Table 4 summarizes the most common primary diagnoses when bronchiectasis was a secondary diagnosis. The most frequent primary diagnosis was COPD exacerbation, 11.3% of cases. The next most frequent was pneumonia with unspecified etiological agents. Other diseases observed were heart failure, pneumococcal pneumonia, asthma and other lung diseases not included in the diagnoses previously considered.

**Table 4:** Most common primary diagnosis in patients discharged with a secondary diagnosis of bronchiectasis in Spain, 2004–2013.

Diagnosis	n	%
Obstructive chronic bronchitis with acute exacerbation	23825	11.3
Pneumonia, organism unspecified	18903	8.9
Acute and chronic respiratory failure	13363	6.3
Acute respiratory failure	11030	5.2
Heart failure	7558	3.6
Other diseases of respiratory system, not elsewhere classified	7541	3.6
Other emphysema	5692	2.7
Obstructive chronic bronchitis with acute bronchitis	5401	2.6
Pneumococcal pneumonia [Streptococcus pneumoniae pneumonia]	4245	2.0
Hemoptysis	3788	1.8
Asthma with acute exacerbation	3319	1.6
Acute bronchitis and bronchiolitis	2768	1.3

**Fig 1:**
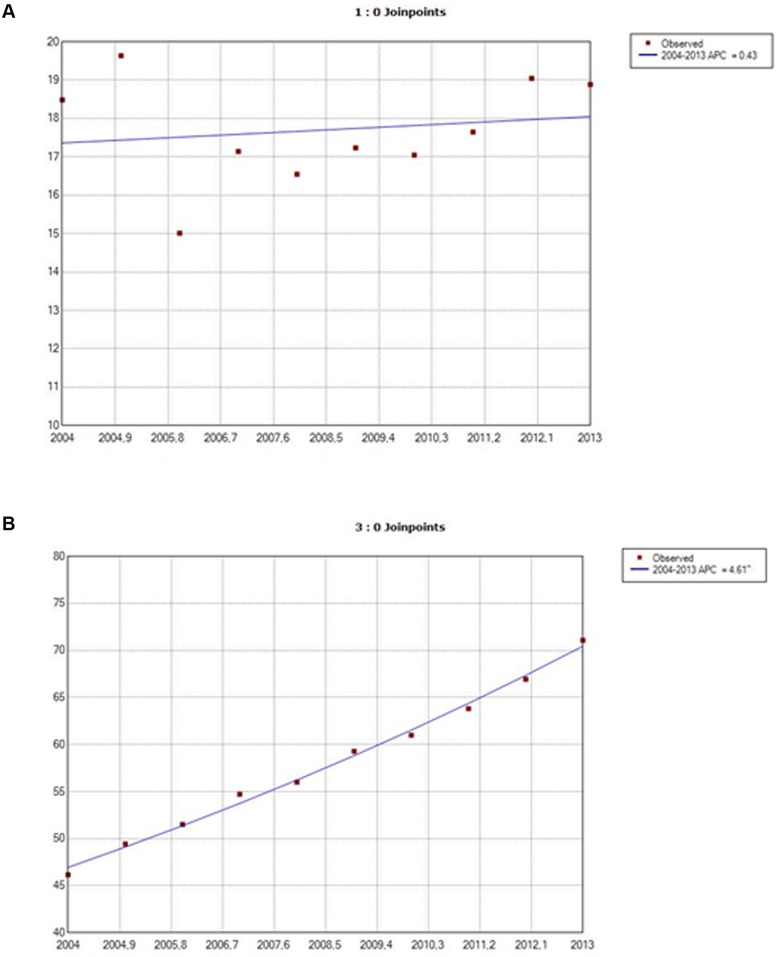
Joinpoint analysis of annual hospitalizations for bronchiectasis in males in Spain, 2004–2013: bronchiectasis as a primary diagnosis (1A), bronchiectasis as a secondary diagnosis (1B). Footnote. APC: annual percent change (based on rates that were sex- and aged-ajusted using the Spanish National Statistics Institute Census projections) calculated using jointpoint regression analysis. Accent: APC is significantly different from zero (two-side, p < 0.05).

**Fig 2:**
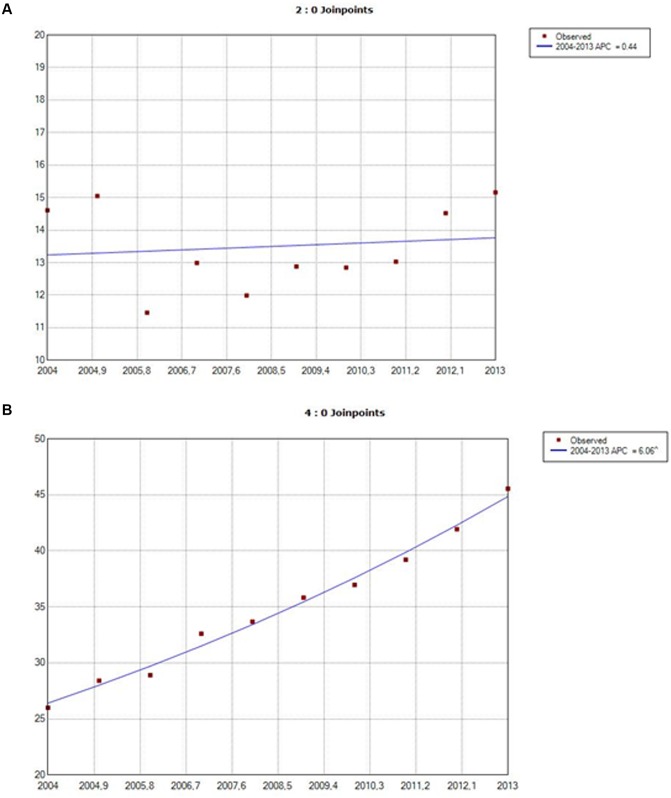
Joinpoint analysis of annual hospitalizations for bronchiectasis in females in Spain, 2004–2013: bronchiectasis as a primary diagnosis (2A), bronchiectasis as a secondary diagnosis (2B). Footnote. APC: annual percent change (based on rates that were sex- and aged-ajusted using the Spanish National Statistics Institute Census projections) calculated using jointpoint regression analysis. Accent: APC is significantly different from zero (two-side, p < 0.05).

Figs 1 and 2 show a joinpoint analysis of the evolution of hospital stays by gender with bronchiectasis as the principal diagnosis and as a secondary diagnosis respectively. As can be seen, the incidence of hospitalization in patients with bronchiectasis as a primary diagnosis increased with an annual percent change (APC) of approximately 0.4% for both sexes (no significant difference). In patients with bronchiectasis as a secondary diagnosis, the APC was higher for women, (6.06%), than for men, (4.61%), and significant for both genders (p<0.05).

Table 5 shows the results of our multivariate analysis of time trends and factors associated with incidence and in-hospital deaths among patients hospitalized with bronchiectasis as the primary or secondary diagnosis in Spain, from 2004 to 2013. After controlling for possible confounders using Poisson regression models, there was a significant decline in incidence from 2004 to 2013 for bronchiectasis as the primary diagnosis. Nevertheless, when bronchiectasis was a secondary diagnosis, we observed an increase in incidence. With regard to in-hospital mortality, after adjusting the logistic regression model, there was a significant decrease in mortality from 2004 to 2013 for bronchiectasis as both the primary and secondary diagnosis. The risk of in-hospital mortality was higher for males, older patients who were non-smokers, patients with Pseudomonas infection, and patients with a higher Charlson comorbidity index.

**Table 5:** Multivariate analysis of trends in incidence and in-hospital mortality (IHM) of bronchiectasis as primary or secondary diagnosis in Spain, 2004–2013.

		Incidence (IRR)		IHM (OR)	
		Bronchiectasis (primary diagnosis)	Bronchiectasis (secondary diagnosis)	Bronchiectasis (primary diagnosis)	Bronchiectasis (secondary diagnosis)
Gender	Male	1	1	1	1
	Female	0.58 (0.57–0.59)	0.46 (0.45–0.47)	0.81 (0.73–0.88)	0.80 (0.77–0.83)
Age groups	0–49	1	1	1	1
	50–64	7.11 (6.87–7.36)	7.34 (7.19–7.49)	1.57 (1.12–2.19)	1.49 (1.33–1.68)
	65–79	36.04 (34.98–37.12)	36.72 (36.09–37.36)	3.00 (2.23–4.04)	1.94 (1.74–2.15)
	≥ 80	71.48 (69.33–73.69)	76.57 (75.22–77.94)	4.98 (3.70–6.71)	3.35 (3.01–3.72)
Pseudomonas aeruginosa infection	No	1	1	1	1
	Yes	0.22 (0.21–0.23)	0.08 (0.08–0.09)	1.44 (1.30–1.60)	1.37 (1.29–1.47)
Tobacco use	No	1	1	1	1
	Yes	0.36 (0.35–0.36)	0.38 (0.37–0.39)	0.66 (0.59–0.74)	0.69 (0.66–0.72)
Charlson index	0	1	1	1	1
	1	0.58 (0.57–0.59)	0.85 (0.84–0.86)	1.54 (1.40–1.69)	1.67 (1.60–1.74)
	≥ 2	0.26 (0.25–0.27)	0.54 (0.53–0.55)	2.01 (1.81–2.25)	2.29 (2.19–2.39)
Year of hospitalizations		0.98 (0.97–0.99)	1.04 (1.03–1.05)	0.97 (0.95–0.98)	0.98 (0.97–0.99)

IRR: Incidence Rate Ratio calculated using multivariate Poisson regression with dependent variable”Year of hospitalizations” and adjusting for all co-variables shown in the table.OR: Odds Ratios calculated using multivariate logistic regression using in hospital mortality as dependent variable” and adjusting for all co-variables shown in the table.

## Discussion

Along the study period, we observed a trend towards a greater incidence of hospitalization, but only when bronchiectasis was codified as a secondary diagnosis, as opposed to when bronchiectasis was the primary diagnosis. Overall, incidence occurred at a rate of 46.48 hospitalizations per 100,000 adults for bronchiectasis as a secondary diagnosis, and 15.53 hospitalizations per 100,000 inhabitants when bronchiectasis was the primary diagnosis. Studies performed in other countries showed an increase in incidence over time [8,9,11,19]. However, hospital admission rates were not uniform. Thus, while in the USA there were 16.5 hospital admissions per 100,000 inhabitants [8], in Hong Kong there were 16.4 hospital admissions per 100,000 inhabitants [20]; in Germany, the annual rate of hospitalization when bronchiectasis was the primary diagnosis decreased to 1.8 per 100,000 inhabitants although when bronchiectasis was either the primary or secondary diagnosis, the rate increased to 9.4 per 100,000 inhabitatns [11].

Regardless of whether or not bronchiectasis was the principal reason for hospital admission, it was more frequently seen among men, in contrast to other data collected in previous studies, in which frequency was greater among women [8,9,11]. However, we observed a downward trend in the proportion of men throughout the study period. This may be due to higher prevalence of tobacco/COPD in males and the patients with COPD and bronchiectasis being more likely to get sick enough to need admission, for COPD and other tobacco related non-pulmonary diseases.

As in previously published studies, incidence increased significantly with age, most significantly in individuals more than 80 years old. This observed increase in the number of hospital admissions related to bronchiectasis may be due to an actual increase in prevalence. However, it might also be explained by other factors, mainly the implementation of the high resolution CT techniques [21], which have allowed for diagnosis of less severe, more silent forms of the disease. Before these diagnostic techniques were available, such cases would have gone not diagnosed, or perhaps wrongly diagnosed as chronic obstructive pulmonary disease (COPD), or as asthma.

Our study found that the most frequent secondary diagnosis, when bronchiectasis was the principal reason for hospitalization, was Pseudomonas aeruginosa infection, as opposed to findings published by other researchers such as Seitz et al [8], who found the most frequent secondary diagnosis to be hemoptysis, followed by Pseudomonas infection and COPD. It is important to remark that in our investigation Pseudomonas infection appeared in 17.37% of patients and increased significantly from 2004 (13.64%) to 2013 (19.46%). As in Germany [11], the primary diagnosis, when bronchiectasis was a secondary diagnosis, was COPD, described in up to 11.3% of cases. Ever since a significant relationship between bronchiectasis and COPD was noted a little more than a decade ago, more and more studies have focused on this association [22,23]. A diagnosis of bronchiectasis as a result of a CT scan was observed to occur in approximately 50% of patients with moderate-severe COPD [24–26]. A study by Martínez-García et al. sought to analyze the risk factors for developing bronchiectasis in a group of patients with moderate-severe COPD. The principal risk factors they found were the presence of a severe obstruction of air flow, a sputum culture positive for a potentially pathogenic microorganism, and a hospital stay during the previous year due to exacerbation. This association between COPD and bronchiectasis should be taken into account in daily clinical practice, since COPD patients with this clinical presentation require a slightly different therapeutic approach, and their prognosis is worse [27].

A less frequent secondary diagnosis in our study was bronchial asthma when bronchiectasis was characterized as the primary diagnosis. In theory, asthma can both precede and follow the appearance of bronchiectasis. Airway obstruction with plugged mucus and a decrease in mucociliary clearance in patients with asthma may predispose these patients to persistent infection and permanent airway damage. On the other hand, ectatic airways may predispose to bronchial hyperreactivity [21]. In other studies, a higher incidence of asthma has been found in bronchiectasis patients [19].

Average hospital stays decreased during the study period, for both bronchiectasis as the main diagnosis for admission, and for bronchiectasis as a secondary diagnosis. This seems reasonable and consistent with the evolution of most respiratory pathologies, for which average hospital stays have become shorter in recent years. However, the cost of hospitalization is lower only for the group with bronchiectasis as the primary diagnosis; and costs have increased for bronchiectasis as a secondary diagnosis. This increase in hospitalization costs when bronchiectasis is the secondary diagnosis could be related to the type of pathology and the severity of the primary diagnosis in each case. As we commented before, one of the main reasons for hospital admission when bronchiectasis is a secondary diagnosis is COPD. Frequently, these patients present more severe disease which would contribute to a greater use of resources and, therefore, an increase in healthcare costs. There are few studies published that analyze the economic burden of bronchiectasis for healthcare systems [4,28], although the data available suggests that bronchiectasis poses a significant economic drain on healthcare resources.

When analyzing patient characteristics, we observed an increase in comorbidity, measured using the Charlson index. This seems reasonable and consistent with the increase in the mean age of patients admitted for bronchiectasis. The number of cases in which Pseudomonas aeruginosa was documented through the study period also rose from 5.96 in 2004 to 6.49% in 2013 (p = 0.016). This was probably due not only to a real increase in the number of Pseudomonas infections and/or colonization, but rather to a greater awareness of the importance of this bacterium, given the implications, both for prognosis and treatment, that Pseudomonas aeruginosa isolation may involve [29,30]. The greater prevalence of Pseudomonas infections in patients hospitalized with bronchiectasis has possibly contributed to the evolution in the costs and length of hospital stay over the study period. Further investigations are needed to assess the impact of this infection including the effect of the use of new diagnosis and therapeutic procedures

As in other studies [8], in-hospital mortality was low and tended to decrease during the study period. Factors associated with an increase in the risk of in-hospital death in our study were age, male gender, non-smoking, Pseudomonas aeruginosa infection, and a higher Charlson comorbidity index.

Pseudomonas aeruginosa infection increased the risk of dying when bronchiectasis was codified as a primary or secondary diagnosis with corresponding OR of 1.44 (95%CI; 1.30–1.60) and 1.37 (95%CI; 1.29–1.47). The negative effect of this infection has been found before [26,28].

The fact that non-smoking could be related to a greater risk of in-hospital mortality might be confusing. However, this may be easier to understand if we bear in mind that patients with the more severe disease generally have quit smoking or never did smoke, precisely because of the severity of their illness. In any case, the data available to date seem to indicate that in-hospital mortality is low. On the other hand, there are few studies available that assess general mortality indexes in bronchiectasis patients [31,32]. Of high interest is a study by Roberts et al [32] which reports on 5,700 deaths from bronchiectasis in Wales and England from 2001 to 2007, showing an annual increase in mortality of 3%. This is important and should raise awareness that the prognosis for bronchiectasis should not be underrated, and that mortality from this cause may even increase in the future. Thus, we should always consider the possibility of bronchiectasis in our diagnosis when a patient visits his doctor with any sort of respiratory complaint.

The current study has recognizable strengths and limitations. The main strength lies in the large sample size and the standardized methodology applied, and constantly maintained during the study period. Nevertheless, our study contains some limitations that should be considered when interpreting our results. First, a potential source of bias comes from the use of ICD-9-CM diagnosis codes to identify patients with bronchiectasis who were hospitalized. The major concern of using disease codes is, of course, the questionable accuracy of bronchiectasis diagnosis. Furthermore, it was not possible to verify that all patients diagnosed with bronchiectasis underwent a CT to confirm the diagnosis. Therefore, bronchiectasis diagnosis could be either underestimated or overestimated. On the other hand, the results shown are limited to the codified variables. Therefore, there are relevant data that cannot be included (for example treatments provided). Such an analysis might have reflected the total impact of the disease on healthcare services in a more realistic manner. Despite these limitations, the CMBD discharge data has the advantage of being mandated by the National Public Health System and includes almost 100% of admissions in Spain [33]. In addition, Spain is a large country with a public health system providing free, comprehensive medical treatment for the entire population, so patients come from a variety of socioeconomic backgrounds, a fact which lends to provide external validity to our results.

In conclusion, data obtained from the present study indicate that there was an increase in the number of hospital admissions with bronchiectasis in Spain during the period 2004 to 2013, but only when the disease was codified as a secondary diagnosis, and the increase was more noticeable in the older population, in particular in individuals older than 80. On the other hand, when bronchiectasis was codified as the primary diagnosis, a decrease in the number of hospital admissions was observed during the study period. This study confirms the strict correlation of bronchiectasis with COPD, with COPD being the most frequent primary diagnosis when bronchiectasis was codified as a secondary diagnosis. The reduction in the average hospital stay and in-hospital mortality during the study period could indicate that management of this pathology has been improving over time.

## Abbreviations

APC: annual percentage of change
COPD: Chronic obstructive pulmonary disease
CMBD: Spanish National Hospital Database, the Conjunto Mínimo Basico de Datos
CT: computerized tomography
GRD: Diagnosis-Related Groups, Grupos Relacionados con el Diagnóstico
ICD-9-CM: International Classification for Disease, 9th revision
IHM: in-hospital mortality
IMV: invasive mechanical ventilation
IRR: incidence rate ratio
LOHS: length of hospital stay
NIV: non-invasive ventilation
OR: odds ratio
SD: standard deviation
